# Topoisomerase II-Mediated DNA Damage Is Differently Repaired during the Cell Cycle by Non-Homologous End Joining and Homologous Recombination

**DOI:** 10.1371/journal.pone.0012541

**Published:** 2010-09-02

**Authors:** Marcelo de Campos-Nebel, Irene Larripa, Marcela González-Cid

**Affiliations:** Departamento de Genética, Instituto de Investigaciones Hematológicas Mariano R. Castex, Academia Nacional de Medicina, Buenos Aires, Argentina; University of Minnesota, United States of America

## Abstract

Topoisomerase II (Top2) is a nuclear enzyme involved in several metabolic processes of DNA. Chemotherapy agents that poison Top2 are known to induce persistent protein-mediated DNA double strand breaks (DSB). In this report, by using knock down experiments, we demonstrated that Top2α was largely responsible for the induction of γH2AX and cytotoxicity by the Top2 poisons idarubicin and etoposide in normal human cells. As DSB resulting from Top2 poisons-mediated damage may be repaired by non-homologous end joining (NHEJ) or homologous recombination (HR), we aimed to analyze both DNA repair pathways. We found that DNA-PKcs was rapidly activated in human cells, as evidenced by autophosphorylation at serine 2056, following Top2-mediated DNA damage. The chemical inhibition of DNA-PKcs by wortmannin and vanillin resulted in an increased accumulation of DNA DSB, as evaluated by the comet assay. This was supported by a hypersensitive phenotype to Top2 poisons of Ku80- and DNA-PKcs- defective Chinese hamster cell lines. We also showed that Rad51 protein levels, Rad51 foci formation and sister chromatid exchanges were increased in human cells following Top2-mediated DNA damage. In support, BRCA2- and Rad51C- defective Chinese hamster cells displayed hypersensitivity to Top2 poisons. The analysis by immunofluorescence of the DNA DSB repair response in synchronized human cell cultures revealed activation of DNA-PKcs throughout the cell cycle and Rad51 foci formation in S and late S/G2 cells. Additionally, we found an increase of DNA-PKcs-mediated residual repair events, but not Rad51 residual foci, into micronucleated and apoptotic cells. Therefore, we conclude that in human cells both NHEJ and HR are required, with cell cycle stage specificity, for the repair of Top2-mediated reversible DNA damage. Moreover, NHEJ-mediated residual repair events are more frequently associated to irreversibly damaged cells.

## Introduction

DNA double strand breaks (DSB) are dangerous lesions threatening the genome stability. DSB are induced from many sources, including oxidative stress, ionizing radiation and chemical compounds [1]. Under non physiological conditions, certain nuclear enzymes, such as Topoisomerase II (Top2), may also generate persistent protein-mediated DNA DSB [2].

Top2 is a ubiquitous enzyme that solves topological problems of the DNA during replication, transcription and chromosome condensation [3], [4]. Top2 catalyzes the interconvertion of topological isomers of DNA through a transient DSB, while remains covalently linked to the 5′ terminus of the DNA, and is followed by double-strand passing and religation [5]. In mammalian cells, there are two isoforms of Top2, α and β, which show similar structural features and catalytic activities [6]. In spite of their similarities, they play different roles in nuclear processes, being Top2α mainly implicated in DNA relaxation/decatenation and segregation [7] and Top2β mostly associated to transcription [8], [9]. Furthermore, the expression of both isoforms is differently regulated, with Top2α levels rising from S phase to M [10], [11] and Top2β remaining constant throughout the cell cycle [12].

Top2 is the main target of several clinically relevant anticancer drugs, such as idarubicin (IDA) and etoposide (ETO) [13], [14]. These agents poison Top2 by stabilizing DNA-Top2 complexes, referred to as cleavable complexes, preventing the religation of the broken ends. Stabilized cleavable complexes are reversible upon drug removal [15]; however, their persistence leads to DSB formation.

In mammals, at least two main DSB repair pathways have evolved, namely non-homologous end joining (NHEJ) and homologous recombination (HR). NHEJ operates by modifying and religating the broken ends regardless of sequence homology; and thus, being potentially mutagenic [16]. NHEJ is controlled by the DNA-PK complex, composed by the DNA-end binding Ku70/Ku80 heterodimer, and the catalytic subunit DNA-PKcs. Additional factors implicated in this pathway include Artemis, Ligase IV, XRCC4 and XLF/Cernunnos [17]–[19]. The activation of DNA-PKcs upon DNA damage involves several autophosphorylation events at serine/threonine residues in two clusterized sites; one of them includes serine 2056 (pS2056DNA-PKcs) [20]–[22]. The autophosphorylation of DNA-PKcs is thought to regulate the kinase activity of the enzyme, as well as the accessibility of other NHEJ components to the DNA ends [23].

In straight contrast, HR is a very precise mechanism, in which a homologous sequence is used as a template to direct the repair process [24]. The HR pathway is mediated by the MRN complex (Mre11/Rad50/Nbs1), RPA, Rad51, Rad52, Rad54, Rad54B, Rad51 paralogs (Rad51B, Rad51C, Rad51D, XRCC2, XRCC3), BRCA1 and BRCA2 [25]–[27]. After an initial end processing by the MRN complex, RPA molecules stabilize the single-stranded DNA stretches. RPA is then replaced by Rad51 to promote the invasion to a homologous template for priming the DNA synthesis [28].

Although Top2 poisons are widely used anticancer drugs, they are often associated to the development of secondary malignancies, particularly therapy-related acute myeloid leukemia (t-AML) [29]. In addition, experimental evidence on knockout mice suggested a tight dependence on Top2β isoform for DSB formation and skin carcinogenesis in response to Top2 poisons [30].

Taking into account the side effects of Top2 poisons, a particular interest has emerged in understanding the repair mechanisms involved in the restoration of Top2-mediated DNA DSB. In this regard, several authors have highlighted the important role of NHEJ in the survival to Top2 poison-induced damage [31]–[33]. On the other hand, HR has been suggested to be functional on Top2 poisons-induced irreversibly DNA damaged cells [32]. It is probable that both DSB repair activities are differently required after either Top2-mediated reversible or irreversible DNA damage, but it still needs to be clarified.

Here we present evidence for an important role of Top2α in the induction of DNA damage by Top2 poisons. In addition, we provide a systematic analysis of the DNA DSB repair activities resulting from Top2-mediated DNA damage in mammalian cells.

## Results

### Top2 poisons induce DSB and cytotoxicity in a Top2α-dependent manner

**Figure 1:**
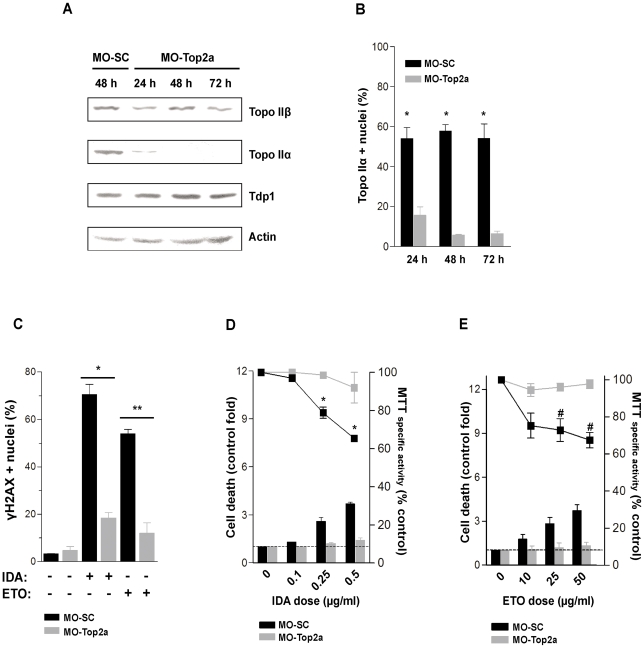
Top2α down regulation and induction of γH2AX and cytotoxicity by Top2 poisons. (A) Western blot and (B) immunofluorescence analysis of Top2α following transfection with 1 µM of MO-Top2a or MO-SC at different times on human fibroblasts. *p = 0.0001 (vs. respective control). (C) At 48 h post-transfection, fibroblasts were treated with IDA (0.01 µg/ml) or ETO (10 µg/ml) for 2 h and analyzed immediately (ETO) or after 4 h (IDA) for the induction of γH2AX by immunofluorescence. *p = 0.0007 (vs. IDA-treated MO-SC); **p = 0.0076 (vs. ETO-treated MO-SC). (D and E) MO-Top2a and MO-SC cells were treated with increasing doses of IDA (D) or ETO (E) for 2 h and analyzed at 36 h by trypan blue exclusion and the MTT assay. * and #p<0.005 (vs. MO-Top2a). All comparisons were performed by the Student *t*-test.

In order to determine the involvement of Top2α in the DNA DSB formation induced by Top2 poisons, we performed knock-down experiments in human fibroblasts with a specific morpholino oligonucleotide targeting Top2α (MO-Top2a) or a standard control (MO-SC). As shown in Fig. 1A, the MO-Top2a caused a substantial downregulation of Top2α at 48 h, but not of Top2β, Tdp1 and actin. This result was confirmed by immunofluorescence analysis, which showed a significant decrease (p = 0.0001) in the number of Top2α positive nuclei at 48 h (Fig. 1B). The proliferation rates after transfection with MO-Top2a were not disturbed during the time course of the experiments (data not shown).

Human fibroblasts transfected with MO-Top2a or MO-SC were treated with 0.01 µg/ml IDA or 10 µg/ml ETO during 2 h, and analyzed for the induction of γH2AX foci (Fig. 1C). This assay revealed that in absence of Top2α, both IDA and ETO induce γH2AX recognizable foci in a small amount of cells (p = 0.0007 and p = 0.0076, respectively).

To elucidate the role of Top2α in the cytotoxicity induced by Top2 poisons, MO-Top2a and MO-SC transfected cells were treated with increasing doses of IDA or ETO and the cell death was determined after 36 h by the trypan blue assay. As shown in Figs. 1D and 1E, increased doses of either drug resulted into an enhanced cell death in MO-SC transfected cells for up to 6.7- and 4.9-fold of control in IDA and ETO treatments, respectively. In contrast, cell death levels increased only up to 1.5- and 1.4-folds respect to controls in MO-Top2a transfected cells, at the highest dose assayed. Concordant results were obtained with the MTT assay. IDA and ETO decreased the metabolic activity of MO-SC transfected cells for up to 65.4%±1.4 (p = 0.0034) and 67.5%±8.2 (p = 0.0008), whereas MO-Top2a transfectants remained with a 92%±11.3 and 97.7%±5.1 of activity, respectively. We conclude, therefore, that Top2α is required for the induction of γH2AX and cytotoxicity after treatment with Top2 poisons in normal human cells.

### Kinetics of DNA DSB repair induced by Top2 poisons

**Figure 2:**
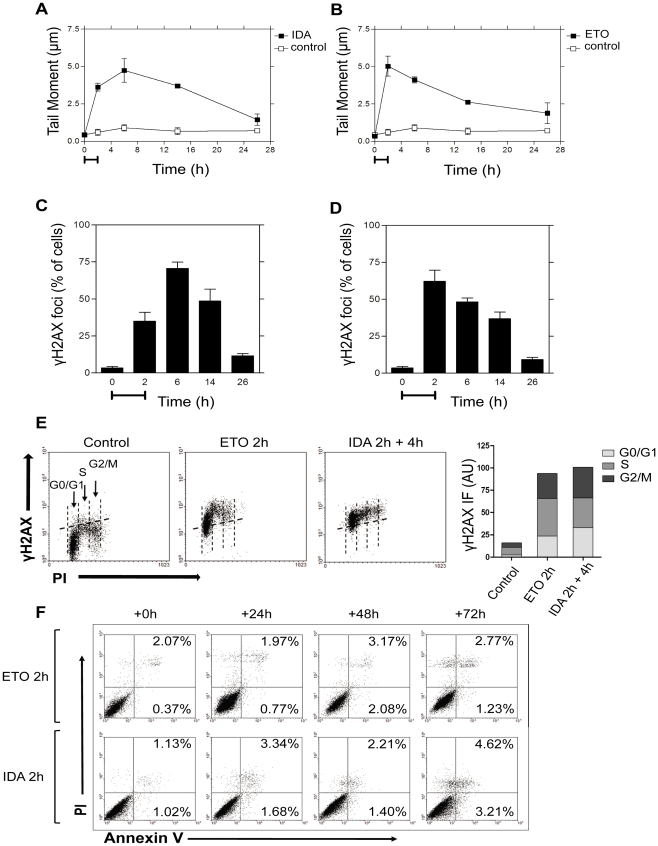
Kinetics of DNA DSB repair induced by Top2 poisons. The kinetics of DNA DSB repair induced by IDA (0.01 µg/ml) or ETO (10 µg/ml) were determined by the neutral comet assay (A and B, respectively) and the resulting γH2AX foci formation confirmed by immunofluorescence (C and D, respectively). Capped lines (A–D) represent the extension of the treatment. (E) Representative flow cytomtery analysis of the γH2AX signals induced by ETO and IDA during the cell cycle (left panels) and the median intensity of fluorescence (IF) per cell cycle phase (right panel). (F) Flow cytometry assessment of apoptotic cell death induced by ETO (10 µg/ml) or IDA (0.01 µg/ml) at 24, 48 and 72 h post-treatment.

To characterize the kinetics of DSB formation and repair, human fibroblast cultures were treated with 0.01 µg/ml IDA or 10 µg/ml ETO for 2 h, and analyzed at different times by the neutral comet assay. As shown in Figs. 2A and 2B, IDA and ETO induced different kinetics of DSB formation, with maximal values at 6 h and 2 h, respectively. They also showed a slow kinetics of repair, with DSB levels remaining above control after 26 h. The evaluation of γH2AX foci formation confirmed these results (Figs. 2C and 2D). The analysis of γH2AX by flow cytometry indicated that both drugs induced DSB in all the stages of the cell cycle (Fig. 2E). The Annexin V analysis (Fig. 2F) for up to 72 h post-treatment showed that DSB induced at doses used in the kinetics study are mostly repairable lesions that do not induce increased cell death.

### NHEJ is involved in the repair of Top2 poison induced DSB

**Figure 3:**
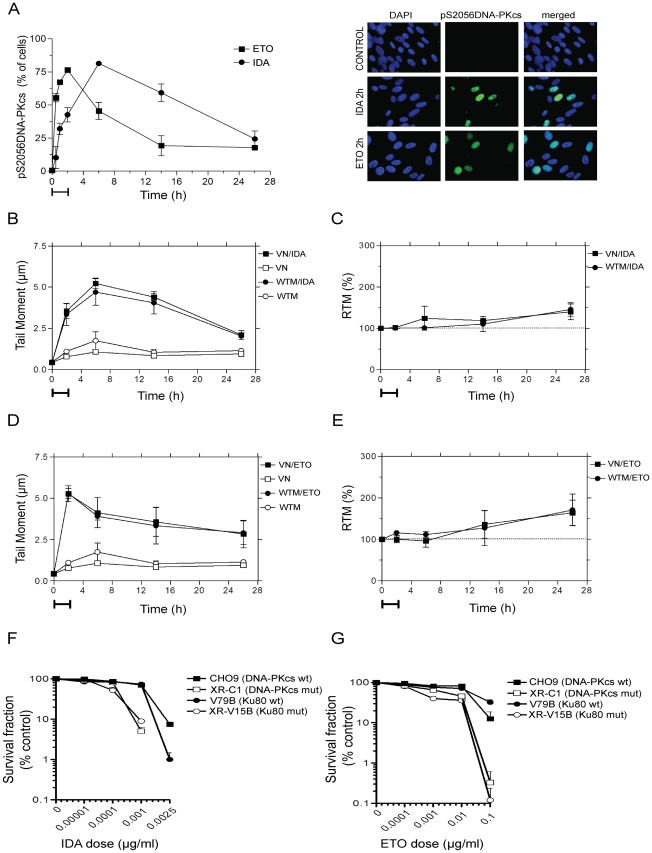
NHEJ is required for the repair of Top2 poisons-mediated DNA DSB. (A) Immunostaining analysis of activated DNA-PKcs in human fibroblasts following the treatment with IDA (0.01 µg/ml) or ETO (10 µg/ml). Representative images are shown in the right panel. (B) Kinetics of DSB repair in human fibroblasts pre-treated with VN (300 µM) or WTM (10 µM) and treated with IDA. (C) Graphical representation of the relative tail moment (RTM) of (B) respect to IDA alone (Fig. 2A). Dotted line represents the tail moment shown by IDA alone as percentage. (D) Kinetics of DSB repair in human fibroblasts pre-treated with VN (300 µM) or WTM (10 µM) and treated with ETO. (E) RTM of (D) respect to ETO alone (Fig. 2B). Dotted line represents the tail moment shown by ETO alone as percentage. Capped lines (A–E) represent the extension of the treatments. Clonogenic survival to IDA (F) or ETO (G) of the wild-type Chinese hamster cell lines CHO9 and V79B, and their NHEJ-mutant counterparts XR-C1 (DNA-PKcs defective) and XR-V15B (Ku80 defective).

To elucidate the participation of NHEJ in the repair of DSB induced by Top2 poisons we evaluated the induction of pS2056DNA-PKcs foci at different times after treatment with IDA or ETO. Fig. 3A shows that both drugs stimulated a rapid accumulation of pS2056DNA-PKcs, with the highest response in accordance with the time of maximal induction of damage. Both treatments showed a small amount of cells with persistent pS2056DNA-PKcs nuclear immunostaining at 26 h.

In order to determine whether the lack of functional NHEJ results into DSB accumulation, we pretreated the human fibroblasts with the DNA-PKcs chemical inhibitors WTM and VN. The doses of WTM and VN assayed diminished the activation induced by ETO of DNA-PKcs by a 49.6% and 58.4%, respectively; with no significant effect on ATM (data not shown).

As shown in Figs. 3B and 3D, both pretreatments did not increase substantially the damage induced by IDA and ETO; but later times resulted into higher levels of damage. Figs. 3C and 3E represent the percentage of damage in pretreated cultures relative to treatment with IDA or ETO alone. After 26 h, VN- and WTM-pretreatments resulted into an accumulation of about 30–50% and 50–60% of damage respect to IDA and ETO alone, respectively. To further probe the involvement of NHEJ in the repair of DSB induced by Top2 poisons, mutant Chinese hamster cell lines were analyzed by the colony formation assay. The experiments showed that XR-C1 (DNA-PKcs mutant) and XR-V15B (Ku80 mutant) cells were hypersensitive to both IDA and ETO (Figs. 3F and 3G). Together, these data demonstrate the important role of NHEJ in the repair of Top2-mediated DNA damage in mammalian cells.

### HR participates in the repair of DSB induced by Top2 poisons

**Figure 4:**
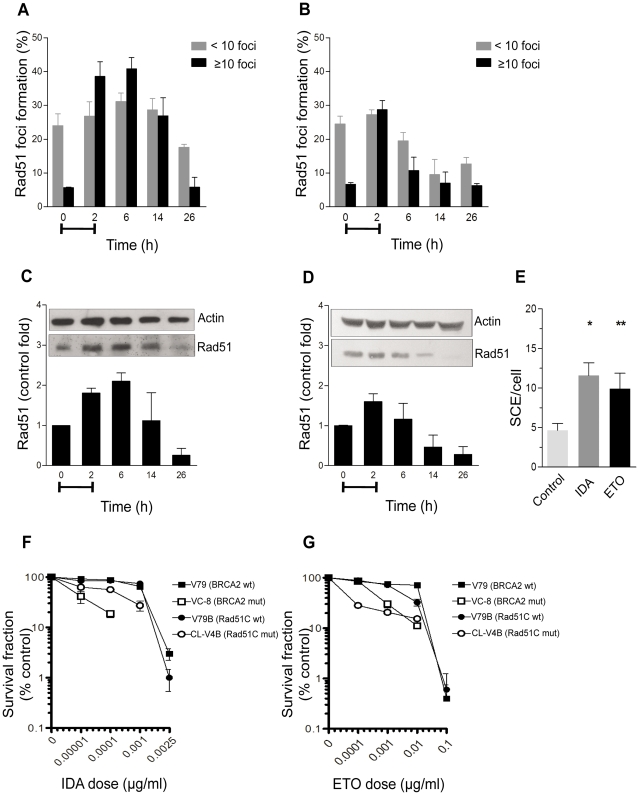
HR is activated in response to DNA DSB induced by Top2 poisons. Rad51 foci are induced in human fibroblasts following treatment with (A) IDA (0.01 µg/ml) or (B) ETO (10 µg/ml). The Rad51 protein levels also increase in response to (C) IDA or (D) ETO. Representative photographs of western blot analysis are shown inside of figures C and D. Data were normalized respect to actin content. Capped lines (A–D) represent the time extension of the treatment. (E) Frequency of sister chromatid exchanges (SCE) in human fibroblasts. (F–G) Clonogenic survival of the wild-type Chinese hamster cell lines V79 and V79B, and their HR-mutant counterparts V-C8 (BRCA2 defective) and CL-V4B (Rad51C defective), following the treatment with IDA or ETO. *p≤0.0055 (vs. control); **p≤0.028 (vs. control). Statistical comparisons were performed by the Student *t*-test.

In order to study the involvement of the HR pathway in the repair of DSB resulting from Top2 poisoning we evaluated the induction of Rad51 foci at different times after treatment with both IDA and ETO in human fibroblasts. Nuclei containing ≥10 foci were scored as positive. Figs. 4A and 4B show that IDA and ETO induced Rad51 foci formation with values that picked up to 6 and 2 h, respectively. Later times resulted into a decreasing number of cells containing Rad51 foci. The analysis of Rad51 protein levels showed similar results (Figs. 4C and 4D). Both drugs enhanced the expression of Rad51 with the highest levels found at times of maximal induction of DNA damage. The decreased levels of Rad51 protein at later times were related to degradation by the 26S proteosome (data not shown).

The events of sister chromatid exchanges (SCE) are believed to result from a Rad51-dependent HR pathway [34]. To test a functional activity of a Rad51-mediated process in response to Top2 poisons, we analyzed the induction of SCE after treatments with IDA and ETO. Fig. 4E shows that both treatments resulted in increased levels of SCE on human fibroblasts.

To further corroborate the participation of HR in the repair of DSB induced by Top2 poisons, we performed colony formation assay in the Chinese hamster cell lines V-C8 (BRCA2 mutant) and CL-V4B (Rad51C mutant). The results showed that mutant cells were hypersensitive to IDA and ETO, as compared to their isogenic wild-type cell lines (Figs. 4F and 4G).

### NHEJ and HR contribute with cell cycle phase specificity to the repair of Top2 poison-induced damage

It has been suggested that DSB repair pathways may be differentially regulated during the cell cycle, with a major activity of NHEJ in G1/early S phase and HR playing a major role on late S/G2 [35], [36].

**Figure 5:**
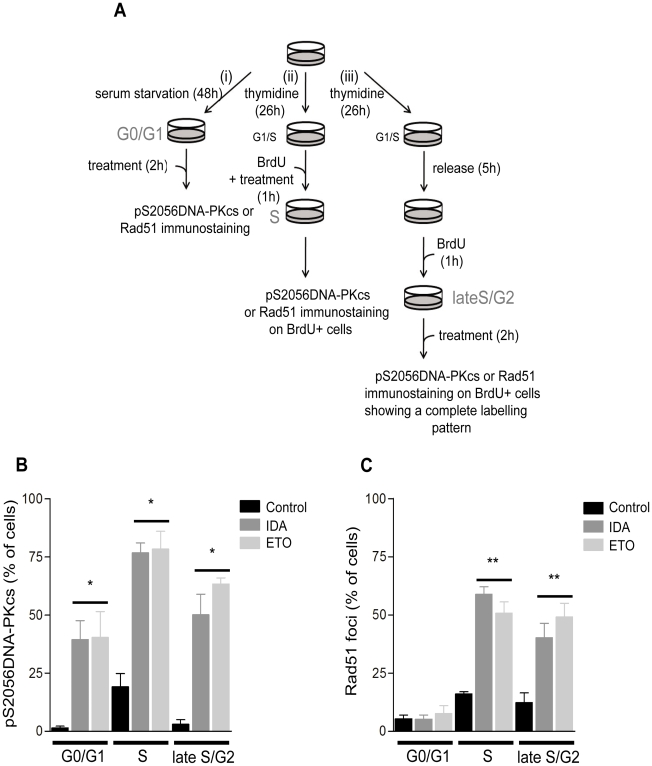
NHEJ and HR are differently required for the repair of Top2-mediated DNA damage in fibroblast cells. (A) Scheme of the synchronization procedure for the analysis of Rad51 and activated DNA-PKcs foci in response to both IDA and ETO treatments. *(i)* Fibroblasts seeded at ∼90% of confluence were subjected to serum starvation during 48 h to obtain a cell population in G0/G1. The media was changed for complete media and the cells were treated with 0.01 µg/ml IDA or 10 µg/ml ETO. Samples were fixed and processed for immunostaining as described in the matherials and methods section. *(ii)* Fibroblasts at 50% of confluence were treated with 2 mM thymidine during 26 h to enrich the cell population in the G1/S border. After washing, complete media containing BrdU (150 µM) and IDA or ETO was added for 1 h. After immunostaining, the scoring was performed on those BrdU+ nuclei. *(iii)* Fibroblasts at 50% confluence were treated with 2 mM thymidine for 26 h. The cells were then rinsed and cultured on fresh complete media for 5 h. After this recovery period, BrdU (150 µM) was added for 1 h and removed by washing with complete media. Finally the cells were incubated with IDA or ETO for 2 h in complete media. Following immunostaining, late S/G2 nuclei were recognized by showing a complete labelling pattern of BrdU. (B) Analysis of activated DNA-PKcs in response to IDA or ETO at different phases of the cell cycle. (C) Rad51 foci formation following IDA or ETO treatments at different stages of the cell cycle. *p≤0.009 (vs. respective control); **p≤0.03 (vs. respective control). Statistical comparisons were performed by the Student *t*-test.

To determine the specific DSB repair activities induced by Top2 poisons throughout the cell cycle, we performed immunofluorescence analysis of pS2056DNA-PKcs and Rad51 on human fibroblast synchronized cultures after treatments with IDA and ETO. A schematic representation of the synchronization conditions and treatments is depicted in Fig. 5A. Under these conditions, S phase cells were scored by the presence of BrdU labelling. In the same way, cells treated during late S/G2 were recognized by their uniformly BrdU-labelled nuclei.

As shown in Fig. 5B, IDA and ETO treatments were able to induce phosphorylation of DNA-PKcs at S2056 in cells at G0/G1 (p≤0.009), S (p≤0.001), and late S/G2 (p≤0.006) phases of the cell-cycle. Whereas the analysis of nuclei containing Rad51 foci revealed that neither IDA nor ETO was able to induce Rad51 foci formation in cells at G0/G1. However, there was a significant induction of Rad51 foci during S phase (p≤0.001), and late S/G2 (p≤0.03) of the cell-cycle (Fig. 5C).

### Persistent DNA damage induced by Top2 poisons results in micronucleated and apoptotic cells containing activated DNA-PKcs

Previous reports have demonstrated that Rad51 foci can be found in micronuclei and apoptotic cells following treatment with different genotoxic agents, including Top2 poisons [37], [38]. Those foci were proposed to result from HR-mediated failed repair events [37]. So far, there is no evidence of residual NHEJ activities associated to micronucleated and early apoptotic cells after a genotoxic insult.

In order to determine whether NHEJ- or HR- residual foci are present in persistently damaged cells, we performed immunofluorescence analysis of pS2056DNA-PKcs and Rad51 on micronucleated and apoptotic cells. As late apoptotic cells show a diffuse staining pattern of activated DNA-PKcs at regions of poor DNA content [39], we looked for Annexin-V labelled cells with pS2056DNA-PKcs nuclear foci remaining on DNA. This staining pattern is very different from that reported by Mukherjee et al. [39]; and therefore, suggestive of residual repair events.

Human fibroblasts were treated for 2 h with increasing doses of both IDA and ETO and analyzed 36 h later. The frequency of γH2AX positive micronucleated cells ranged from 86 to 90% for either treatment throughout the doses assayed (data not shown).

**Figure 6:**
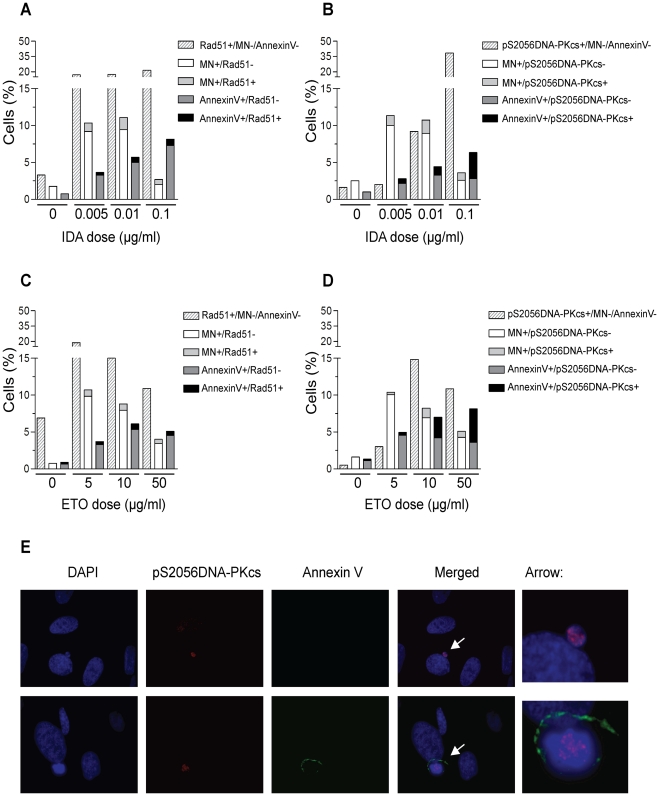
Residual repair events are associated to persistently DNA damaged cells by Top2 poisons. Human fibroblasts treated with increasing doses of IDA (A) or ETO (C) showing Rad51 residual repair events on micronuclei and apoptotic cells. Active DNA-PKcs remaining foci are found in irreversibly DNA damaged cells following the treatments with IDA (B) and ETO (D) at different doses. (E) Representative images of micronuclei (top panel) and apoptotic cells (bottom panel) containing activated DNA-PKcs foci following treatment with ETO.

Figs. 6A and 6C show the frequency of micronucleated and apoptotic cells containing Rad51 foci after increasing dose of IDA and ETO, respectively. The frequency of micronucleated cells containing Rad51 foci induced by IDA was low and represented from 11.2% to 14.7% of the micronucleated population. Similarly, the values obtained after treatment with ETO ranged from 8.2% to 13.6%. Apoptotic cells containing Rad51 foci represented 10–11% of the apoptotic population for all the increasing doses of both IDA and ETO treatments.

The analysis of micronucleated cells containing pS2056DNA-PKcs showed frequencies ranging from 11.6% to 27.8% after IDA treatment (Fig. 6B), while 5% to 16.7% were found after ETO treatment (Figs. 6D and 6E, top panel). The scoring of apoptotic cells containing pS2056DNA-PKcs foci showed a higher increase, with frequencies ranging from 40.6% to 63% for IDA (Fig. 6B), and 14.3% to 55.6% for ETO (Figs. 6D and 6E, bottom panel).

## Discussion

Topo2α levels and tumor proliferation rates are considered, among others, predictive factors for the effective implementation of Top2 poisons in chemotherapy. In addition, several breast cancers have shown TOP2A amplification and overexpression [40]; which makes them particularly susceptible to Top2 poisons.

Top2 poisons are widely exploited in cancer treatment, with therapy-related secondary tumors being a major unresolved problem. In order to improve chemotherapy, it is necessary to understand better how cells do respond to Top2-mediated damage.

In this study, we pursued two major aims. First, to determine the role of Top2α in the generation of DNA DSB by Top2 poisons in normal human cells. Secondly, to analyze the DSB repair pathways responsible for the restoration of the genome integrity following the Top2-mediated DNA damage.

Top2 poisons stabilize the cleavable complexes irrespective of Top2 isoform. However, it has been suggested that Top2β is mainly implicated in the induction of DSB, while Top2α is essential for citotoxicity [30]. Although the role of Top2β in the generation of DSB was attributed to the preferential degradation by the proteasome pathway of Top2β-DNA cleavable complexes, recent evidence demonstrated that both isoforms are targets for the proteasome and that alternative processing mechanisms may also be involved [41]. We show here that Topo2α is actively implicated in the induction of γH2AX and citotoxicity by Top2 poisons on normal human fibroblasts. In this regard, two recent reports are in line with our results: (i) in vitro studies on mice tumoral cell lines showed that Top2α knock down resulted into diminished DNA damage signals and increased resistance to doxorubicin [42]; and (ii) NK314, a novel Top2α-specific poison was shown to induce DSB efficiently in human tumoral cells [43].

Our data indicate that DNA DSB induced by subtoxic concentration of Top2 poisons may occur at any stage of the cell cycle and are mostly reversible lesions. To investigate the involvement of NHEJ in the repair of Top2-mediated damage, we use different approaches. We show that DNA-PKcs is rapidly activated in response to DNA damage induced by Top2 poisons. In addition, chemical inhibition of DNA-PKcs results into an extensive accumulation of DSB in human cells. Furthermore, Ku80- and DNA-PKcs-deficient Chinese hamster cell lines display a hypersensitive phenotype, as assessed by clonogenic survival. In close agreement with our data, it has been reported that the DNA-PKcs deficient V3-3 Chinese hamster cell line was hypersensitive to m-AMSA [44].

On the other hand, a previous study on chicken DT40 cell lines suggested a major role for a DNA-PKcs-independent NHEJ pathway in the repair of ETO-induced damage [45]. This pathway is well represented in V(D)J recombination, where the blunt end ligation that results in signal joints can take place in the absence of DNA-PKcs, while coding joints which requires nucleolytic end processing steps cannot [46], [47]. Thus, it is assumed the requirement of DNA-PKcs in NHEJ relies on the DNA ends configuration. Our data demonstrate that in mammalian cells DNA-PKcs-dependent NHEJ is strongly activated and responsible for the repair of an important fraction of the DNA DSB induced by Top2 poisons. However, we cannot rule out any contribution of a DNA-PKcs-independent NHEJ mechanism. Although most of the randomly occurring DSB generate incompatible DNA termini, the exact nature of the DNA ends resulting from Top2-mediated damage is not well understood. In this regard, the observed activation of DNA-PKcs may also reflect that incompatible DNA ends are generated.

To determine whether the conservative HR is also implicated in the repair of Top2-mediated damage, we analyzed several end points. In human fibroblasts Rad51 protein levels, Rad51 nuclear foci and SCE are substantially increased following Top2-mediated DSB. Similarly, an increased expression of Rad51 protein in response to ETO was recently reported in colorectal adenocarcinoma cell lines [48]. Furthermore, BRCA2- and Rad51C-mutant Chinese hamster cells show hypersensitivity to the Top2 poisons. In agreement with our findings, it was reported an increased sensitivity to Top2 poisons of XRCC3-deficient Chinese hamster cells [44], BRCA1- and BRCA2-mutant human tumoral cells [49], and a middle sensitivity of *rad54^−/−^* and *rad52^−/−^* chicken DT40 cell lines [32], [50].

Since NHEJ and HR may operate differently throughout the cell cycle, we analyzed pS2056DNA-PKcs and Rad51 foci formation on human fibroblasts subjected to cell cycle synchronization. Our data show that DNA-PKcs-dependent NHEJ is activated by Top2-mediated damage in all cell cycle phases, while Rad51-dependent HR is induced in S and late S/G2 phases. The spontaneous autophosphorylation of DNA-PKcs on S phase cells was previously reported [22] and is believed to result from physiologically relevant amounts of replication-associated DNA DSB.

These results are consistent with the NHEJ and HR activities reported on mammalian cells after ionizing radiation induced damage [51] and I-SceI endonuclease-mediated DSB on chromosomally-integrated reporter systems [52].

Due to the middle sensitivity to ETO displayed by *ku70^−/−^rad54^−/−^* double mutants compared with the *ku70^−/−^* simple mutant DT40 cells, Adachi et al. [32] proposed that HR-mediated DSB repair may be a cytotoxic repair pathway for Top2-mediated DNA damage. This was suggested assuming that Top2 poisons induce an irreversible type of DNA damage, especially at high doses, which could be a substrate for HR. Previous studies have demonstrated that following genotoxic stress, micronuclei and apoptotic cells containing Rad51 and RPA residual foci are induced [37], [38]. Therefore, we evaluated whether NHEJ- or HR-mediated residual repair events result from persistent DNA damage induced by Top2 poisons. The levels of micronuclei and apoptotic cells containing pS2056DNA-PKcs remaining foci rise with the increasing doses of the drugs. However, Rad51-mediated residual repair events remain at similar low levels. Taking into account the strength of the HR repair response, it is unlikely the low level of cells showing HR-mediated residual foci may support a cytotoxic effect of HR. On the other hand, the increased levels of pS2056DNA-PKcs remaining foci on persistently DNA damaged cells may represent a constant effort of the cell to restore unrepaired DNA fragments, which could lead to mutations. It is noteworthy that non-homologous-mediated end joining of DNA DSB was proposed to reverse apoptosis, albeit at low frequency, leading to the development of chromosomal translocations, particularly those linked to t-AML [53], [54].

Overall, our data support an important role of Top2α for the induction of DNA DSB and cytotoxicity by Top2 poisons in normal human cells. Moreover, both NHEJ and HR repair activities are required to restore the genome integrity after Top2-mediated DNA damage with cell cycle phase specificity. Finally, the finding of activated DNA-PKcs residual foci on persistently DNA damaged cells opens important questions about how long the NHEJ components may continue operating to reverse the DNA damage-induced apoptotic cell death and how the proper degradation of NHEJ factors is controlled under this scenario. Future studies should shed light on these issues and determine the relevance of these mechanisms in the development of cancer.

## Materials and Methods

### Reagents, Cell cultures and treatments

IDA (CAS no. 58957-92-9; Pharmacia y Upjohn), bromodeoxyuridine (BrdU; CAS no. 59-14-3; Sigma) and vanillin (VN; CAS no. 121-33-5; Sigma) were dissolved in bidistilled water. ETO (CAS no. 33419-42-0; Sigma) and wortmannin (WTM; CAS no. 19545-26-7; Sigma) were dissolved in DMSO.

The human foreskin fibroblast cell line PTP, kindly provided by Dr MI Tous (Servicio de Cultivo de Tejidos, Depto Virología, ANLIS “CG Malbrán”, Buenos Aires, Argentina), was grown in Minimum Essential Medium supplemented with 10% FBS and 2 mM L-glutamine. Mutant hamster cell lines deficient in NHEJ (XR-C1, XR-V15B) and HR (CL-V4B, V-C8) with their parental cell lines (CHO9, V79B and V79) were used. CHO9, XR-C1 and XR-V15B were grown in F-12 medium supplemented with 10% FBS and 2 mM L-glutamine. V79, V79B, CL-V4B and V-C8 were cultured in Mc Coy's 5A medium (without hypoxanthine and thymidine) supplemented with 10% FBS. The hamster cell lines were generously kindly provided by Prof Dr MZ Zdzienicka and Dr W Wiegant (Leiden University, Leiden, The Netherlands). All the cell cultures were incubated at 37°C under a 5% CO_2_ humidified atmosphere.

Human fibroblasts were treated with 0.01 µg/ml IDA or 10 µg/ml ETO for 2 h, unless otherwise specified. Chinese hamster cell lines were treated for 20 h with different doses of IDA or ETO, as specified in the figures.

### Transfections and Top2α depletion

Exponentially growing human fibroblasts were transfected with 1 µM of morpholino antisense oligonucleotide targeting top2α mRNAs (MO-Top2a; 5′-TTTACAGGCTGCAATGGTGACACTT-3′; Gene Tools) or a standard control (MO-SC; 5′-CCTCTTACCTCAGTTACAATTTATA-3′; Gene Tools) using 6 µM of Endo-Porter reagent (Gene Tools) in complete medium, as specified by manufacturers. The efficiency of Top2α knockdown was analyzed by immunofluorescence and western blot at different time points.

### Cell death analysis

For the assessment of cell death, three methodologies were used:

- *Trypan Blue exclusion assay*. After 48 h of transfection, human fibroblasts growing on 48-well plates were treated for 2 h with different doses of IDA or ETO and analyzed 36 h later by adding 100 µl of 1% trypan blue solution into each well containing 100 µl of complete medium. The percentage of cell death was determined, under inverted microscope, in 300 cells from at least 8 different fields in 3 independent experiments.
- *MTT assay*. Human fibroblasts (2.5×10^3^) were cultured in 96-well plates, transfected with MO-Top2a and grown for 48 h. Then the cells were treated with the DSB inducer drugs for 2 h and grown for other additional 36 h. The MTT reagent [3-(4,5-dimethylthiazol- 2-yl)-2,5-diphenyltetrazolium bromide; Sigma] was added for colorimetric reaction up to a final concentration of 1 mg/ml. After 2 h incubation at 37°C, cell lysis and MTT solubilization with DMSO, samples were assayed in an ELISA reader at 570 nm. The MTT specific activity was determined and expressed as the percentage of control from 3-5 independent experiments.
- *Annexin V*. For flow cytometry analysis of apoptosis, the Annexin V-FITC Apoptosis Detection Kit (Calbiochem) was used according to manufacturer's instructions.

### Neutral Comet assay

The human fibroblasts were mock or pretreated with WTM (10 µM) or VN (300 µM) for 0.5 h before the exposure to IDA or ETO and harvested after different periods of time. WTM and VN were present in the media during all the culture time. We performed the neutral single cell gel electrophoresis as described by Angelis et al. [55] with slight modifications. Briefly, cell suspensions were embedded in agarose and deposited in microscope slides. The slides were incubated 2 h at 4°C in lysis solution (2.5 M NaCl, 100 mM EDTA, 10 mM Tris, pH 10, 10% DMSO, 1% Triton X-100) followed by 3 washes in neutralizing buffer (0.4 M Tris-HCl, pH 7.5). Electrophoresis was carried out for 20 min at 20 V in 0.5% TBE buffer (pH 8). Slides were then stained with a 20 µg/ml ethidium bromide solution. Images from 50 nucleoids/sample/time-point were scored in 3 independent experiments. The analysis of the tail moment (TM) was performed with the CASP software.

### Immunofluorescence

Human fibroblasts were grown in coverslips, fixed with 2% paraformaldehyde and permeabilized with 0.25% Triton X-100 at room temperature. After blocking (3% BSA, 0.25% Triton X-100 in PBS) for 1 h, cells were incubated for 2 h with mouse anti Top2α (1∶300; Chemicon; clone KiS1), mouse anti γH2AX (1∶500; Upstate; clone JBW301), rabbit anti Rad51 (1∶250; Santa Cruz) or rabbit anti pS2056DNA-PKcs (1∶400; a generous gift from Dr. BP Chen, University of Texas Southwestern Medical Center, Dallas, USA). Appropriated secondary fluorescein isothiocyanate (FITC) or Texas red-conjugated anti-rabbit (1∶250, Vector Laboratories) or anti-mouse (1∶250, Vector Laboratories) antibodies were used. DNA was counterstained with 4,6-diamidino-2-phenylindole (DAPI; Vector Laboratories) containing mounting medium.

### Cell synchronization and immunolabelling

Human fibroblasts were synchronized in the different cell cycle stages and treated with IDA or ETO as depicted in Figure 5A. Cultures were fixed and permeabilized with 2% paraformaldehyde/0.5% Tween 20 in PBS for 20 min. Samples (S- and G2-synchronized cells) were treated with 2N HCl for 30 min and then neutralized with 0.1 M Borax buffer (sodium tetraborate, pH 8.5). Immunofluorescence was performed as described previously. To detect S- and G2-phase nuclei a monoclonal anti BrdU antibody (1∶200; Invitrogen) was used. One hundred nuclei were scored for the presence of Rad51 or pS2056DNA-PKcs foci. Three independent experiments were carried out for each treatment.

### Flow cytometry

For flow cytometry analysis of γH2AX, cellular suspensions were fixed with paraformaldehyde 1% in PBS and permeabilized in 0.25% Triton X-100 in PBS, and then processed for immunolabelling as previously described. After incubation with the secondary antibody, samples were treated with 200 µg/ml RNAse A and 20 µg/ml propidium iodide (PI) in PBS for 0.5 h at 37°C. Thirty-thousand cells per sample were evaluated and the median γH2AX intensity of fluorescence per cell cycle phase was calculated as described by Huang et al. [56].

### Western blot

Whole cell extracts were prepared in RIPA buffer (50 mM Tris-HCl pH 7, 150 mM NaCl, 1% NP-40, 0.5% sodium deoxycolate and 0.1% SDS) containing a cocktail of protease inhibitors and the protein concentrations were determined by the Bradford method [57]. Fifty µg of total proteins were separated on 8% SDS-PAGE and transferred onto PVDF membranes. After blocking overnight in 0.2%Tween20/PBS containing 5% non-fat dry milk, membranes were incubated for 2 h with mouse anti Top2α antibody (1∶500), mouse anti Top2β (1∶500; Santa Cruz), goat anti Tdp1 (1∶400; Santa Cruz), rabbit anti Rad51 (1∶1000) or mouse anti actin (loading control, 1∶1000, Calbiochem) in blocking buffer. Appropriated HRP-conjugated anti mouse, anti rabbit or anti goat antibodies (1∶10000) and the ECL kit (Amersham) were used for protein detection. Following autoradiography, the bands were analyzed with the Gel Pro analyzer software (Media Cybernetics).

### Sister chromatid exchanges

Human fibroblasts were treated for 2 h with 0.01 µg/ml IDA or 10 µg/ml ETO and then, incubated in the presence of 10 µg/ml BrdU for two complete rounds of replication (50 h). The average frequency of SCE was determined from the analysis of 30 metaphases during the second cycle of division in three independent experiments.

### Colony formation assay

Chinese hamster cells were plated at low density (300–1000 cells/dish), allowed to attach for 4 h, and treated with different concentrations of IDA or ETO for 20 h. The cells were then washed with PBS and cultured in fresh medium for 7–10 days. After this growth period, the cells were rinsed with PBS, fixed with methanol, and stained with crystal violet. Only colonies containing more than 50 cells were scored. Survival fraction was calculated as the ratio of colonies in drug-treated cultures compared with control cultures and expressed in percentages. Three independent experiments were carried out for each end point.

### Rad51 and pS2056DNA-PKcs foci detection on micronucleated and apoptotic cells

Human fibroblasts growing on coverslips were treated with increased concentrations of IDA or ETO for 2 h. The cells were washed and cultured in complete media for other additional 36 h. To detect apoptotic cells we incubated the preparations with FITC-annexin V, rinsed in washing buffer (10 mM HEPES/NaOH, pH 7.4; 140 mM NaCl; 5 mM CaCl_2_), and simultaneously fixed and permeabilized in 1% paraformaldehyde containing 0.25% Triton X-100/PBS at room temperature for 20 min. Samples were then immunolabelled with anti Rad51 or anti pS2056DNA-PKcs as previously described. The frequency of micronucleated and apoptotic cells containing either Rad51 or pS2056DNA-PKcs foci was determined by analyzing at least 1000 cells per sample.
